# Supplementary material for: Identifying the Risk Regions of Wild Boar (Sus scrofa) Incidents in China
Source: Animals (Basel). 2023 Oct 12;13(20):3186. doi: 10.3390/ani13203186 (PMC10603701; doi:10.3390/ani13203186)
Supplement: Supplementary file 1 [file animals-13-03186-s001.zip › animals-2555569-supplementary.pdf]

Table S1. Risk space area of the whole country and each province.

Region	Low (km²)	Sub-low (km²)	Medium (km²)	Sub-high (km²)	High (km²)	Sub-high and high (km²)	Sub-high and high (%)
National	6343719	1143437	979365	780566	393524	1174090	12.18
Shaanxi	28704	31915	25031	44102	75847	119950	58.34
Sichuan	238716	78785	75086	65082	28331	93413	19.22
Hubei	12885	37064	54019	55512	26420	81932	44.07
Jiangxi	5127	24826	62421	62849	11677	74526	44.65
Shanxi	21675	36016	25457	33272	40280	73552	46.94
Hunan	8494	39237	93267	61993	8808	70801	33.43
Guizhou	463	26631	79795	62925	6353	69279	39.33
Yunnan	99061	140863	86911	55554	11711	67265	17.07
Henan	27680	52505	24922	31697	30196	61893	37.06
Shandong	31836	38025	24808	30430	30701	61131	39.24
Gansu	312711	28472	23986	26823	33808	60631	14.24
Zhejiang	7365	17244	23880	34413	22598	57011	54.04
Anhui	18039	42932	24987	31416	22726	54142	38.65
Guangxi	8038	94111	91590	38843	5018	43860	18.46
Liaoning	14932	50822	43185	31279	7783	39062	26.39
Chongqing	779	11167	34352	29998	6107	36104	43.81
Fujian	5796	40447	41777	26496	9484	35981	29.02
Guangdong	15729	73967	58649	26729	4652	31381	17.46
Jiangsu	30320	42965	17045	10207	6662	16869	15.74
Hebei	89715	51438	33157	11906	2584	14490	7.67
Ningxia	49258	10454	2888	3045	753	3799	5.72
Beijing	615	5746	7472	2175	402	2577	15.70
Jilin	127804	43121	14690	1537	249	1785	0.95
Shanghai	331	2178	2150	1406	276	1682	26.53
Tibet	1216121	10386	1514	355	24	379	0.03
Qinghai	703814	15927	2311	174	75	248	0.03
Inner Mongolia	1153476	27444	1930	150	0	150	0.01
Tianjin	7994	3123	700	150	0	150	1.25
Heilongjiang	431643	40684	626	48	0	48	0.01
Hainan	12126	22585	690	0	0	0	0.00
Xinjiang	1662475	2356	70	0	0	0	0.00
